# Practical Notes on Remedies

**Published:** 1906-06-09

**Authors:** 


### Practical Notes on Remedies.

### On the Use of Aloin in Chronic Constipation.

The two drugs most commonly taken by people who suffer from chronic constipation are cascara and aloin. Cascara sagrada is often stated to have an advantage over aloin in that it acts not merely on the colon, as does aloin, but on the small intestine as well. Very many people find its use very satisfactory; they take 2 grains of the solid extract every night or every other night, with the result that the bowels act next morning. The great drawback to its use, however, is that the drug gradually loses its effect—*i.e.* if it is taken for many months it is generally found that increasing doses have to be employed. For instance, one patient, in the course of eighteen months, had to increase the dose from 2 grains to 8 grains. This, too, is not an isolated or exceptional case; one is continually coming across them. This serious defect of cascara is not possessed by aloin. The contrary is often the case, especially if the drug is administered as stated below. The late Dr. Spender, of Bath, was one of the first to advocate the use of aloin in cases of chronic constipation. He advised that 1 grain of aloin, in pill, should be given at dinner-time. If this does not act next day 2 grains should be given on the following evening; and so on until the bowels act. No drastic saline or purgative should be given if the bowels do not act, or otherwise one will have to begin over again. If on any one day the bowels act more than once, or if a loose action takes place, the dose should be diminished by one pill. This is an excellent plan of treatment. Its only defect is that the "steps" are too great. A better plan is the following. Have the pills made up in $\frac{1}{4}$-grain doses, thus:

℞

| | |
|---|---|
| Aloini ... ... ... ... ... | gr. $\frac{1}{4}$ |
| Ext. bellad. ... ... ... ... | gr. $\frac{1}{16}$ |
| Ext. nux vom. ... ... ... ... | gr. $\frac{1}{16}$ |
| Gingerin. ... ... ... ... | gr. $\frac{1}{16}$ |

M. ft. pil.

Begin by giving six of these. If this does not act increase the dose by one pill each night until the bowels act. Then keep on with this dose until there is either a loose action or two actions of the bowels, and then take one pill less—and so on. The variations of dose are thus much less, and it will be found that patients can leave off one pill at a time much more easily than with the bigger pills. This plan has been frequently found to answer most admirably. Thus, one old lady a year ago was taking seven pills each night; now she gets on comfortably with two, and occasionally three, pills. Nervous patients may be a little alarmed at having to take so many pills at a time, but a little explanation soon satisfies them. A word may be added as to the other ingredients of the pill and their quantities. The belladonna and nux vomica aid the action of the aloin, and the gingerin tends to prevent griping. Their amounts cannot safely be increased beyond those stated, as so many pills have often to be administered at first.

